# Structural Insights into the Impact of the M142I Mutation in Monkeypox Virus G9 Protein on Subcomplex Formation Revealed by AlphaFold 3 Modeling

**DOI:** 10.3390/molecules31091466

**Published:** 2026-04-28

**Authors:** Xudong She, Yuan Liang, Linqing Wang, Yifan Lin, Xuenan Zhang, Li Zhu, Qinghua Wu, Weiwei Xiao, Chengsong Wan, Kexin Xi, Wei Zhao, Chenguang Shen, Bao Zhang, Jianhai Yu

**Affiliations:** BSL-3 Laboratory (Guangdong), Guangdong Provincial Key Laboratory of Tropical Disease Research, Key Laboratory of Infectious Diseases Research in South China, School of Public Health, Southern Medical University, No. 1023, South Shatai Road, Baiyun District, Guangzhou 510515, China

**Keywords:** monkeypox virus, entry fusion complex, APOBEC3, AlphaFold 3, subcomplex formation, M142I mutation, protein–protein interaction

## Abstract

The membrane fusion process, mediated by the entry fusion complex (EFC) of the monkeypox virus (MPXV), is crucial for host cell invasion. Apolipoprotein B mRNA Editing Catalytic Polypeptide-like 3 (APOBEC3)-driven mutation bias is a key factor in MPXV’s adaptive evolution during its global spread. However, how these mutations affect the structure and function of EFC proteins remains poorly understood. To address this, we performed genomic mutation analysis on globally circulating MPXV clades Ib and IIb, combined with protein monomer, binary, and quaternary complex structure modeling based on AlphaFold 3 and experimental validation by ELISA. We first delineated the mutational spectra of all 11 EFC proteins, revealing that although EFC proteins in clade Ib are highly conserved, lineage IIb B exhibits extensive APOBEC3-driven mutations and the G9 M142I mutation is identified as a lineage-associated APOBEC3-type mutation of lineage IIb B. Structural predictions revealed that while the M142I mutation does not alter G9 monomer folding, it induces a conformational shift in the G9/A16 subcomplex. Furthermore, within the predicted G9/A16/A56/K2 quaternary complex, this mutation enlarges the interfacial gap and reduces docking stability between the G9/A16 subcomplex and A56/K2. Experimental validation demonstrated that the M142I mutation significantly reduces the binding affinity of G9 for A16 and impairs the recruitment of A56/K2 to the quaternary complex, confirming the computationally predicted mechanism of interface destabilization. These findings highlight a dynamic interplay between APOBEC3-driven evolution and EFC protein structure, demonstrating that the M142I mutation alters EFC complex assembly dynamics and may shift the regulatory balance of the membrane fusion system. These structural changes provide molecular insights into MPXV lineage differentiation, though direct functional assays are required to determine the net effect on viral entry efficiency.

## 1. Introduction

Monkeypox is a zoonotic infectious disease caused by the monkeypox virus (MPXV). In May 2022, an unprecedented mpox outbreak rapidly escalated into a global public health crisis, prompting the World Health Organization to declare mpox as a Public Health Emergency of International Concern in July 2022 [1] and again in August 2024 following the emergence of clade Ib in Africa [1,2,3]. Unlike mpox outbreaks before 2022, which primarily occurred in East, Central, and West African countries, the current global outbreak has a wider geographical spread, faster transmission speed, and significantly increased frequency of human-to-human transmission. These factors are accelerating the rapid adaptive evolution of MPXV, affecting its infectivity and pathogenicity [4,5].

MPXV belongs to the genus *Orthopoxvirus* in the *Poxviridae* family. Like other orthopoxviruses, MPXV produces two types of enveloped virions, mature virions and extracellular virions, both of which require the same entry fusion complex (EFC) for membrane fusion to enter host cells [6]. The EFC consists of 11 proteins, namely G9, A16, J5, A21, A28, H2, G3, L5, F9, L1, and O3, all of which are highly conserved among orthopoxviruses. The absence or inhibition of any single EFC protein leads to membrane fusion defects, affecting viral infection [7,8]. EFC proteins can form stable subcomplexes through interactions to achieve the membrane fusion process. Three subcomplexes have been identified: A28/H2, A16/G9, and G3/L5. Notably, individual EFC proteins contribute at different stages of the membrane fusion process; for example, G9, A16, A21, H2, G3, F9, and O3 appear to function during the hemifusion initiation stage, while A28, L1, and L5 function during the post-hemifusion stage [9]. Currently, the extracellular domain structures of almost all orthopoxvirus EFC proteins (except J5 and O3) and the binding structures of subcomplexes have been resolved [10,11,12]. However, reports on the structural characteristics of MPXV EFC proteins and their molecular interaction mechanisms remain very limited.

Mutation bias driven by Apolipoprotein B mRNA Editing Catalytic Polypeptide-like 3 (APOBEC3) is considered a key driver of mutations in the currently globally spreading MPXV [13]. These mutations have accumulated extensively in MPXV after 2022 and show continuous temporal persistence [14]. This accumulation can be traced back to as early as 2016 and formed the MPXV clade IIb lineage B, which caused widespread global transmission in 2022 [15]. Such mutations were also observed in the MPXV clade Ib that emerged in Africa in 2024 [16,17], suggesting that APOBEC3 may also drive mutations in MPXV EFC proteins, thereby affecting the viral membrane fusion process and potentially altering viral infectivity and host adaptation. However, although multiple MPXV pathogenicity or immune-related proteins, such as B16R, N2L, G10R, and B21R, have been reported to have APOBEC3-like mutations affecting their functions [18,19], the mutation status of EFC proteins remains poorly understood. Nevertheless, adaptive mutations in EFC proteins may be related to the transition of mpox from endemic local outbreaks to global spread, as recent studies in vaccinia virus (VACV) have shown that adaptive mutations in the G9 protein of EFC can affect the initiation of membrane fusion [20,21].

In this study, we adopt a multi-scale research strategy from population genomics to atomic scale structure prediction and biochemical validation, with the objective of elucidating the specific mutational spectra of 11 EFC proteins during the evolution of MPXV through genomic mutation analysis on the globally rapidly spreading MPXV clades Ib and IIb. Furthermore, by using AlphaFold 3 to predict the structures of mutated individual proteins and their subsequently formed subcomplexes, combined with ELISA-based binding assays to experimentally validate the predicted alterations in protein–protein interactions, we aimed to reveal the potential molecular basis by which specific mutations in EFC proteins affect the MPXV membrane fusion process.

## 2. Results and Discussion

### 2.1. Mutational Spectrum Analysis of MPXV EFC Proteins

**Figure 1:**
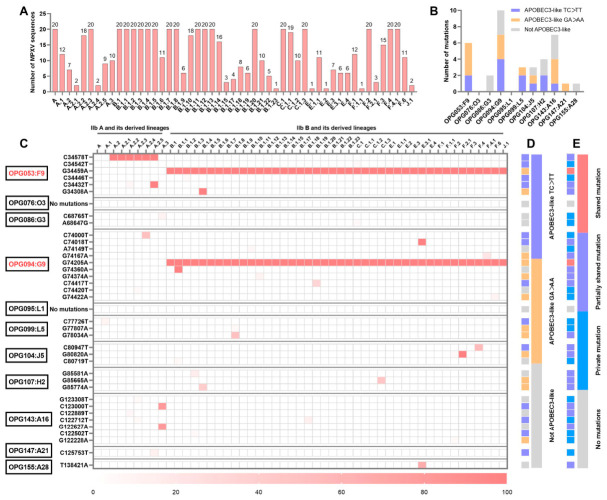
Mutational analysis of 11 EFC proteins in the MPXV clade IIb with APOBEC3-driven characteristics. (**A**) Lineage distribution of the 633 MPXV sequences included in the MPXV-IIb dataset; (**B**) Frequency of APOBEC3 mutations in the 11 EFC proteins in the MPXV-IIb dataset; (**C**) Summary of all mutation site information for the 11 EFC proteins in the MPXV-IIb dataset; (**D**,**E**) APOBEC3 mutation types and sharing types in each lineage for each mutation in the 11 EFC proteins in the MPXV-IIb dataset.

This study created two MPXV sequence datasets: MPXV-I (*n* = 100) (Supplementary Data S1 and S2) and MPXV-IIb (*n* = 633) (Supplementary Data S3 and S4). The SNPs extracted from these two datasets exhibited distinct mutational characteristics. Among the 11 EFC proteins in MPXV-Ib, only F9 and G9 proteins had 3 and 1 unique APOBEC3-like mutations, respectively, while the other proteins were completely conserved compared to MPXV clade Ia. This result is highly consistent with previous reports indicating that although APOBEC3-like mutations have been observed in MPXV clade Ib, no temporally persistent shared mutations have been identified [16,17,22]. In contrast, compared to the MPXV lineage IIb A, APOBEC3-driven mutations were identified in various EFC proteins of lineage IIb B and its derived lineages, including all three types of mutations: those shared by all lineage IIb B, those shared by some derived lineages, and those unique to single strains (Figure 1).

We focused on summarizing the mutational spectrum of the MPXV-IIb dataset (Supplementary Data S5), which encompassing all 51 derived lineages of clade IIb. Compared to the MPXV lineage IIb A, 9 of the 11 EFC proteins encoded by MPXV circulating after 2022 underwent mutations, with 7 proteins exhibiting APOBEC3-driven mutational characteristics (Figure 1A,B), specifically annotated as TC > TT or GA > AA mutation types [13]. Notably, unlike other proteins, which primarily had unique mutations in single MPXV sequences, both F9 and G9 had a shared APOBEC3 mutation that could completely distinguish the IIb A and IIb B lineages: F9’s G34459A mutation (amino acid mutation: P78S) and G9’s G74205A (amino acid mutation: M142I). In the globally circulating lineages after 2022, such as IIb A.2, A.3, A.4, A.5, and IIb B derived lineages, multiple partially lineage-shared APOBEC3 mutations were also isolated, suggesting that these protein mutations may be related to MPXV’s global spread (Figure 1C–E). However, according to existing research reports, F9 interacts weakly with other EFC proteins and may function only as a peripheral component rather than a core component in membrane fusion [6,23,24]. In contrast, G9 plays a critical role in the hemifusion initiation stage, and its binding to A16 to form a subcomplex can directly initiate membrane fusion [9,20,21]. Therefore, we aimed to further clarify the impact of the G9 protein’s M142I mutation on subcomplex formation.

### 2.2. Protein Structure Prediction and the Subcomplex Modeling of the MPXV G9 Protein M142I Mutation

Based on the core role of the G9/A16 subcomplex in EFC-mediated membrane fusion, recent studies have found that the G9 protein is more prone to mutations, while A16 maintains conservation. The G9 protein’s mutations may be key to affecting subcomplex formation and initiating membrane fusion [20,21]. However, unlike other enveloped viruses, MPXV encodes fusion inhibitory proteins A56 and K2. During infection, A56/K2 form a complex on the cell surface, directly targeting the G9/A16 subcomplex to prevent superinfection and syncytium formation, thereby controlling the timing and localization of viral fusion. Recent studies have shown that viruses lacking A56 or K2, or those with G9 mutations, produce large syncytia at neutral pH during infection, affecting viral infectivity [25,26]. This suggests that the G9 protein M142I mutation identified in this study may not only affect G9/A16 subcomplex formation but also influence the interaction between A56/K2 and G9/A16.

**Figure 2:**
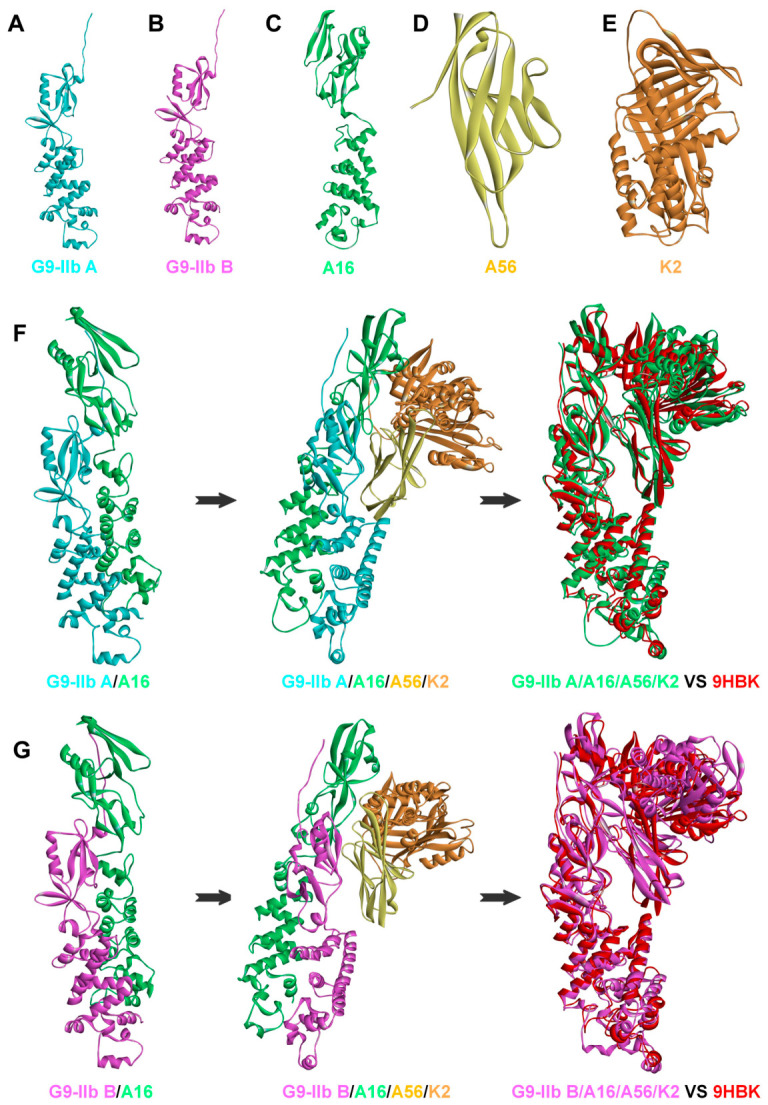
Three-dimensional structure prediction of the mpxv G9 protein and modeling of complex formation. (**A**–**E**) Represent the three-dimensional structure models of the G9-IIb A, G9-IIb B, A16, A56, and K2 proteins, respectively; (**F**,**G**) Represent the structural modeling process of G9-IIb A and G9-IIb B forming subcomplexes with A16, further forming quaternary complexes with A56/K2, and finally superimposed with the homologous complex of VACV.

To investigate the potential molecular structural basis of the G9 protein M142I mutation between MPXV clades IIb A and IIb B, we first predicted the three-dimensional structures of the G9-IIb A, G9-IIb B, A16, A56, and K2 proteins. Subsequently, we completed the binary complex docking structure predictions for G9-IIb A/A16 and G9-IIb B/A16, as well as the quaternary complex structure modeling for G9-IIb A/A16/A56/K2 and G9-IIb B/A16/A56/K2. The results showed that the overall folding of the five protein monomers was clear, with well-defined secondary structure features, indicating good structural rationality (Figure 2A–E). Structural alignment of predicted monomers with the experimental VACV structures (9HBK) yielded RMSD values of 1.495 Å (G9-IIb A), 1.537 Å (G9-IIb B), 1.315 Å (A16), 1.101 Å (A56), and 1.55 Å (K2), confirming high accuracy of the de novo predictions. Inspection of the residue 142 neighborhood (5Å radius) revealed no steric clashes with adjacent side chains (D141, N143, C145) in either variant (Figure S1). Cross-method validation using Protenix, Boltz-2, and OpenFold3 confirmed high structural concordance with AlphaFold3, with Cα RMSD values ranging from 0.679 Å to 1.638 Å (Table S1). Specifically, for the G9 proteins, the three alternative methods yielded RMSD values of 0.849–1.17 Å (G9-IIb A) and 0.778–1.621 Å (G9-IIb B) relative to AlphaFold3, confirming that the monomer folds are robustly reproduced across diverse prediction architectures. These cross-method validations, combined with the existing experimental structural alignment against VACV 9HBK (Figure S1A–E), provide independent evidence supporting the reliability of our predicted monomer structures.

The final modeled quaternary complexes, when superimposed with the recently resolved homologous quaternary complex of VACV (PDB ID: 9HBK) [26], demonstrated high consistency between the two predicted quaternary complex models and 9HBK, with overall topological structures and key functional domain arrangements largely overlapping. Particularly in the core backbone region, the carbon backbone trajectories of the two showed high alignment, exhibiting satisfactory superposition effects (Figure 2F,G). This suggests that the quaternary complex compact conformations modeled in this study based on AlphaFold 3 possess high credibility and precision.

### 2.3. Molecular Structural Analysis of the Impact of the MPXV G9 Protein M142I Mutation on Subcomplex Formation

**Figure 3:**
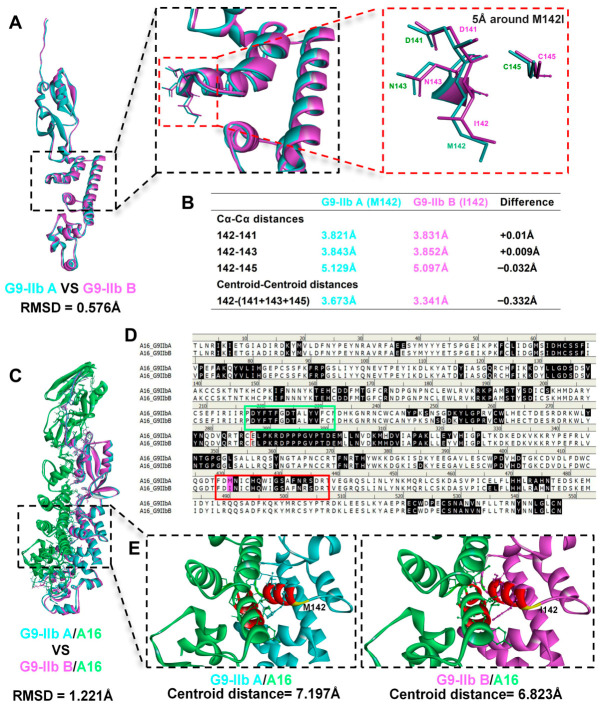
Structural comparison of G9 monomers and G9-A16 binary complexes. (**A**) Superposition of G9-IIb A (cyan) and G9-IIb B (magenta) monomer structures with C-termini oriented downward. Left panel shows the global view with overall Cα RMSD (0.576 Å); middle and right panels display progressive zooms (black and red dashed boxes) of the 5Å radius region around residue 142, showing sidechains of M142 (cyan) and I142 (magenta) with neighboring residues (D141, N143, C145); (**B**) The C α - C α distance between residue 142 of G9-IIb A and G9-IIb B monomers and other individual residues within a 5 Å radius region, as well as the centroid to centroid distance between the residue 142 and cluster formed by all other residues within a 5 Å radius region; (**C**) Structural superposition of G9-IIb A/A16 (cyan/green) and G9-IIb B/A16 (magenta/green) binary complexes. The global alignment (left) displays the overall Cα RMSD (1.221 Å); the dashed box indicates the zoomed region covering the docking interface; (**D**) Amino acid sequence alignment of G9/A16. Black highlighting indicates interface residues; red boxes highlight G9 residues adjacent to position 142; green boxes highlight A16 residues interacting with the G9 142-neighborhood; magenta highlighting represents the G9 M142I mutation; (**E**) Close-up views of the M142I neighborhood at the G9-A16 docking interface. Interface residues are colored red; the centroids of the G9 and A16 interface residue sets are indicated, with distances annotated (7.197 Å for G9-IIb A/A16; 6.823 Å for G9-IIb B/A16).

To elucidate the potential impact of the M142I mutation, structural differences in the G9 protein were analyzed in three states, namely monomer, subcomplex, and quaternary complex, through superposition and comparative analysis. Superposition of the G9-IIb A and G9-IIb B monomer structures revealed high overall folding consistency with an RMSD of 0.576 Å, indicating significant structural homology. However, local analysis showed that while the main-chain conformations near the M142I mutation site remained largely consistent (Cα-Cα distance variations ≤ 0.032 Å), there were subtle differences in side-chain orientation, reflected by a 0.332 Å contraction in centroid-to-centroid distances between residue 142 and its neighbors (D141, N143, C145), leading to a conformational shift in this region without perturbing the backbone architecture (Figure 3A,B).

Structural superposition of the G9-IIb A/A16 and G9-IIb B/A16 binary complexes revealed an overall Cα RMSD of 1.221 Å (Figure 3C), indicating preserved global docking geometry but localized interface perturbations. Sequence analysis identified the docking interface residues (Figure 3D). Detailed inspection of the M142I neighborhood (Figure 3E) revealed a reduction in the centroid distance between the G9 and A16 interface residue sets (from 7.197 Å to 6.823 Å), suggesting localized reorientation of the interface. However, quantitative analysis showed that while total buried surface area increased by 36.3 Å^2^ (+1.5%) and hydrogen bonds increased from 23 to 28, the G9-A16 overall centroid distance increased by 0.314 Å (Table 1). Notably, the G9 nonpolar contact surface area increased by 39.66 Å^2^ (+6.0%), reflecting the enhanced hydrophobicity of isoleucine. These structural alterations were characterized by increased interface contacts but expanded overall inter-protein distance and altered local geometry.

**Figure 4:**
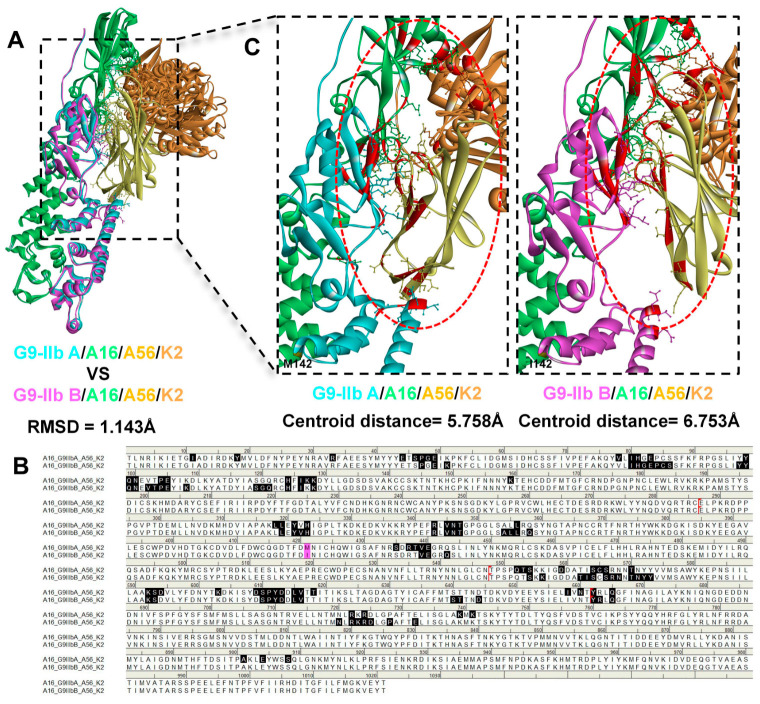
Structural modeling of G9/A16/A56/K2 quaternary complexes and impact of M142I mutation on complex assembly. (**A**) Superposition of G9-IIb A/A16/A56/K2 (cyan/green/yellow/orange) and G9-IIb B/A16/A56/K2 (magenta/green/yellow/orange) quaternary complexes, showing the overall Cα RMSD (1.143 Å); (**B**) Sequence alignment showing the docking interface residues (black highlighting) between the G9/A16 subcomplex and A56/K2 complex; magenta highlighting represents the G9 M142I mutation; red dashed box represents the delimiter between different protein sequences; (**C**) Close-up views of the docking interface between G9/A16 and A56/K2. Interface residues are colored red; the centroid distances between the G9/A16 interface residue set and the A56/K2 interface residue set are annotated.

Quaternary complex modeling revealed that while the overall architecture was preserved (Cα RMSD = 1.143 Å, Figure 4A), the M142I mutation induced distinct assembly modes between the two variants. Notably, the M142I site is located within the G9/A16 subcomplex and does not directly participate in the docking interface with A56/K2 (Figure 4B). However, the mutation induced a more “open” conformation characterized by an enlarged interfacial gap: the centroid distance between the G9/A16 and A56/K2 interface residue sets increased from 5.758 Å to 6.753 Å (Figure 4C), with the overall G9/A16-A56/K2 centroid distance increasing by 1.535 Å (+3.4%, Table 1). Quantitative analysis further revealed that despite an increase in total buried surface area (+24.3%), the hydrogen bond count decreased from 9 to 6 (−33.3%), indicating compromised interface quality. These structural alterations indicate that the G9 M142I mutation propagates conformational changes from the G9/A16 core to the distal A56/K2 interface.

### 2.4. Experimental Validation of Computational Predictions by ELISA

**Figure 5:**
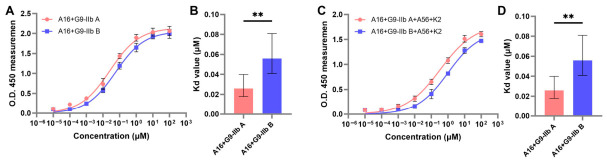
Experimental validation of the M142I mutation impact on EFC complex formation. (**A**,**B**) Binding affinities of G9-IIb A and G9-IIb B for A16 in binary complexes; (**C**,**D**) The effect of M142I mutation in quaternary complexes; (**A**,**C**) Dose–response curves represent nonlinear regression fits to the specific binding model with variable Hill slope. Data points represent mean ± SD of three independent experiments performed in triplicate; (**B**,**D**) Kd_app values showing ~2.2-fold increase for binary (*p* = 0.0048) and ~2.9-fold increase for quaternary (*p* = 0.0088) complexes. Data are mean and 95%CI from three independent experiments. Statistical significance was determined by extra sum-of-squares F test comparing Kd_app values. ** *p* < 0.01.

To validate the computational predictions, direct binding ELISA was performed to assess the impact of the M142I mutation on binary and quaternary complex formation. The M142I substitution significantly reduced the affinity of G9 for A16 (*p* < 0.01), with Kd_app values increasing from 0.026 μM (95% CI: 0.018–0.040) for G9-IIb A to 0.056 μM (95% CI: 0.041–0.081) for the G9-IIb B (Figure 5A,B). This effect was amplified at the quaternary level: recruitment of A56/K2 to the A16/G9 subcomplex was impaired 2.9-fold in the M142I-containing complex (Kd_app = 1.002 μM, 95% CI: 0.588–2.143) compared to G9-IIb A-containing quaternary complex (Kd_app = 0.342 μM, 95% CI: 0.214–0.621; *p* < 0.01) (Figure 5C,D). These results confirm that the M142I mutation destabilizes EFC protein interactions in a cumulative manner.

Collectively, these biochemical data demonstrate that the M142I mutation functionally compromises both core subcomplex formation and regulatory complex assembly, supporting the AlphaFold 3-predicted mechanism of interface destabilization and providing experimental validation for the structural basis of viral specific mutation. Our findings align with previous studies in VACV demonstrating that adaptive mutations in G9 enable escape from A56/K2-mediated fusion inhibition. Hong et al. demonstrated that the G9 H44Y mutation lowers the pH threshold for fusion activation, suggesting that G9 mutations may mimic acid-induced conformational intermediates [20]. Similarly, Cotter et al. isolated VACV mutants with substitutions near the N terminus of G9 (H44Y, H44R, and Y42C) that overcome A56/K2-imposed entry restrictions and induce syncytium formation at neutral pH [21]. Chiu et al. further identified critical residues in VACV G9 essential for membrane fusion and complex assembly through systematic mutagenesis [25]. Notably, Meola et al. recently resolved the cryo-EM structure of the A16/G9/A56/K2 quaternary complex, revealing that A56/K2 recognizes the N-terminal domains of both A16 and G9 [26].

In our study, the M142I mutation presents a mechanistic paradox regarding membrane fusion regulation. On the one hand, reduced A56/K2 recruitment to the quaternary complex could theoretically alleviate fusion inhibition, potentially facilitating viral entry. On the other hand, the significantly reduced G9/A16 binding affinity may impair assembly of the core entry fusion machinery. Without direct membrane fusion or viral entry assays, we cannot definitively determine whether the net effect of M142I is enhanced, reduced, or altered fusion efficiency. The safest interpretation is that this mutation disrupts the precise regulatory balance of the EFC system, potentially affecting the timing or localization of membrane fusion during viral entry, rather than simply enhancing or suppressing fusion activity.

## 3. Limitations

While this study integrates computational predictions with biochemical validation, several limitations remain. The binding measurements represent apparent dissociation constants (Kd_app) from solid-phase ELISA, not definitive solution-phase values. The immobilized A16 and sequential assembly (A16 → G9 → K2 → A56) may introduce avidity effects, orientation constraints, and surface artifacts. Additionally, N-terminal tags (6 × His for G9, FLAG for A16, Strep-tag II for K2) may interfere with binding given that A56/K2 recognizes N-terminal domains; however, identical tag design for both G9 variants ensures valid relative comparison. While relative affinities between variants remain valid under identical conditions, absolute values may differ from orthogonal methods (BLI, SPR, ITC).

The ELISA experiments demonstrate that the M142I mutation reduces binding affinity in binary and quaternary complexes, but future research should further validate the functional consequences of this mutation through in vitro membrane fusion assays and viral infection experiments to directly assess its impact on viral entry efficiency. Additionally, although our AlphaFold 3-predicted quaternary complex models exhibit high structural consistency with the published VACV homologous complex (PDB: 9HBK), cryo-electron microscopy or X-ray crystallography will be essential to resolve the true atomic structure of the MPXV G9/A16/A56/K2 complex and precisely map the conformational alterations induced by the M142I mutation. We also acknowledge that the M142I mutation was identified through large-scale genomic analysis of circulating MPXV strains, and while our multi-scale modeling strategy—from monomer structure to subcomplex and quaternary complex formation—provides a robust structural framework, the dynamic nature of protein–protein interactions in the context of authentic viral membranes warrants further investigation through single-particle tracking or live-cell imaging approaches.

## 4. Materials and Methods

### 4.1. Mutation Analysis of MPXV EFC Proteins

Genetic diversity sequence datasets for MPXV clades I and IIb were created based on the NCBI Virus public database (https://www.ncbi.nlm.nih.gov/labs/virus/vssi/#/, accessed on 6 January 2026) to analyze single nucleotide polymorphisms (SNPs) in MPXV that rapidly spread globally after May 2022. Specifically, as of 6 January 2026, 3894 complete MPXV genomic sequences were retrieved from the NCBI Virus database using the search criteria “Virus: Monkeypox virus” and “Nucleotide sequences: Complete.” For the 3036 clade IIb MPXV sequences, 20 sequences were randomly selected from each lineage using a random number method to form the MPXV-IIb dataset (Supplementary Data S3 and S4), encompassing all 51 derived lineages of clade IIb. If fewer than 20 sequences were available, all were included, resulting in a final dataset of 633 MPXV sequences. For the 829 clade I MPXV sequences, 50 sequences each from clades Ia and Ib were randomly selected using a random number method to form the MPXV-I dataset (Supplementary Data S1 and S2). The system evolutionary trees of both datasets were constructed using NextClade v2.14.1 online server (https://clades.nextstrain.org/, accessed on 2 February 2026) to confirm the lineage of each MPXV sequence (Figure S2). The 11 EFC protein mutation site information for each sequence in the datasets was extracted using MPXV-M5312_HM12_Rivers (lineage IIb A, GenBank Accession No. NC_063383.1) and Zaire_1979-005 (clade Ia, GenBank Accession No. DQ011155.1) as reference strains to analyze the specific mutational spectra of globally spreading MPXV clades Ib and IIb.

### 4.2. Protein Monomer Structure Modeling

For structural consistency with experimental templates (PDB: 9HBK) and subsequent ELISA validation, extracellular domain sequences (with signal peptides and transmembrane regions removed) of A16, A56, G9-IIb A, G9-IIb B, and K2 proteins were used for AlphaFold3 prediction to obtain three-dimensional structural information of the proteins (Supplementary Data S6). To eliminate potential template bias from existing experimental structures in the Protein Data Bank (PDB), all monomer structure predictions were performed using AlphaFold3 (https://github.com/google-deepmind/alphafold3, accessed on 2 February 2026) in de novo prediction (template-free) mode, where structural models were generated based solely on amino acid sequence information and multiple sequence alignment (MSA) without utilizing PDB templates. MSAs were generated using the default genetic databases (UniProt, MGnify, and BFD) integrated within the server pipeline. AlphaFold3 structure predictions were performed via the web server. For each protein or complex, five models were generated and the representative model with the highest Ranking score (model_0) was selected for downstream analysis. Model confidence metrics including pLDDT scores, PAE matrices, and ipTM values are provided in Supplementary Data S8. After model generation, to validate the accuracy of AlphaFold3 de novo predictions, structural alignment was performed by superimposing predicted monomers onto the corresponding protein chains in the experimentally determined vaccinia virus complex structure (PDB: 9HBK) using Discovery Studio 2016. RMSD values for Cα atoms were calculated to assess prediction accuracy. To validate prediction robustness, three independent de novo methods (Protenix: https://protenix-server.com/, accessed on 23 April 2026; Boltz-2: https://app.tamarind.bio/boltz, accessed on 23 April 2026; OpenFold3: https://app.tamarind.bio/openfold, accessed on 23 April 2026) were employed in template-free mode for all five monomers. Cα structural alignments against AlphaFold3 predictions were performed using Discovery Studio 2016 (Table S1, Supplementary Data S9).

### 4.3. Binary Complex Docking Structure Prediction

Building on the monomer structure predictions, to elucidate the direct interaction modes between proteins, binary complex structure predictions were further performed for G9-IIb A/A16 and G9-IIb B/A16. The AlphaFold3 multi-chain input mode was used for protein–protein complex structure modeling. In the binary complex predictions, each interacting pair was modeled as an independent system, focusing on the binding interface characteristics and conformational matching relationships between the two protein chains. After model generation, the two representative complex structures output by AlphaFold3 were compared to analyze the impact of the G9 protein’s M142I mutation on subcomplex formation. For binary complexes, interface analysis focused on G9-A16 interactions. Interface residues were defined as those with any heavy atom within 5Å of the docking partner. Quantitative metrics including buried surface area (BSA), polar/nonpolar contact distributions, hydrogen bonds, salt bridges, and π-interactions were calculated using the Analyze Protein Interface module. The G9-A16 centroid distance was measured between the geometric centers of the respective chains to assess overall docking compactness.

### 4.4. Quaternary Complex Structure Simulation

Building on the binary interaction analysis, to further reveal the overall assembly mode under multi-protein collaboration, two quaternary complex structures, G9-IIb A/A16/A56/K2 and G9-IIb B/A16/A56/K2, were constructed and predicted. Unlike binary complex predictions, quaternary complex modeling emphasizes the spatial collaborative arrangement and interface competition/synergistic effects of multiple protein chains within the same system. Therefore, the AlphaFold3 multi-chain overall input strategy was used for complex structure prediction. After model generation, the two representative complex structures output by AlphaFold3 were superimposed and compared with the latest resolved VACV homologous quaternary complex (PDB ID: 9HBK) to assess the biological rationality of the models from the perspectives of overall conformational consistency and key structural domain spatial arrangement. For quaternary complexes, interface analysis extended to the G9/A16-A56/K2 interface. Interface residues were defined as those between the G9/A16 subcomplex and A56/K2 complex. Centroid distances were calculated between (i) the entire G9/A16 subcomplex and A56/K2 complex, and (ii) the respective interface residue sets specifically, to quantify inter-subcomplex spacing and “openness” of complex assembly. All predicted structural models (monomers, binary and quaternary complexes) are deposited in PDB format as Supplementary Data S7.

### 4.5. Experimental Validation of Computational Predictions by ELISA

Recombinant MPXV EFC proteins were expressed for experimental validation. The proteins were expressed in 293F cells using the pCAGGS-MGS vector(Provided by Professor Shen Chengguang from Southern Medical University) with the following N-terminal tags: 6 × His for G9-IIb A, G9-IIb B, and A56; FLAG for A16; and Strep-tag II for K2. Proteins were purified by affinity chromatography (Ni-NTA for His-tagged proteins, Anti-Flag Affinity gel for A16, and Streptavidin resin for K2), dialyzed against PBS, and stored at −80 °C until use.

Binding affinities were determined by direct ELISA as apparent dissociation constants (Kd_app). These Kd_app values represent surface-based binding parameters and should not be interpreted as definitive solution-phase dissociation constants. Kd_app values were calculated by nonlinear regression fits to the specific binding model with variable Hill slope using GraphPad Prism v9.5.1. Statistical significance was determined by extra sum-of-squares F test comparing Kd_app values. ** *p* < 0.01. Briefly, FLAG-tagged A16 (2 μg/mL) was coated onto 96-well plates overnight at 4 °C. After blocking and washing, serial dilutions of His-tagged G9-IIb A or G9-IIb B were added and incubated for 1 h at 37 °C. Bound G9 was detected using HRP-conjugated anti-His antibody, followed by TMB substrate development and absorbance measurement at 450 nm. For quaternary complex assessment, stepwise sequential assembly (A16 → G9 → K2 → A56) was performed on immobilized A16-coated plates: following G9 incubation, K2 and A56 were added sequentially, with detection of complex-bound K2 using anti-Strep-tag II-HRP.

## 5. Conclusions

This study systematically revealed the specific mutational spectra of 11 EFC proteins in the globally circulating MPXV clades Ib and IIb through genomic mutation analysis, identifying the G9 protein M142I mutation as a key APOBEC3-driven mutation distinguishing lineages IIb A and IIb B. Structural predictions based on AlphaFold 3 revealed that this single amino acid mutation, M142I, in the G9 protein may propagate local conformational fluctuations to the G9/A16 subcomplex interface, thereby altering the overall assembly stability of the G9/A16/A56/K2 quaternary complex. Experimental validation by ELISA confirmed that the M142I mutation significantly reduces the binding affinity of G9 for A16 in binary complexes and impairs the recruitment of A56/K2 to the quaternary complex, supporting the computationally predicted mechanism of interface destabilization. These findings provide a structural basis for understanding how APOBEC3-driven mutational patterns affect EFC protein interactions. The M142I mutation alters the assembly dynamics of both binary and quaternary complexes, potentially shifting the regulatory balance of the membrane fusion system. However, direct functional validation through membrane fusion assays and viral infection experiments is required to determine the net effect on viral entry efficiency and to understand the epidemiological implications of this lineage-associated mutation in the global spread of MPXV lineage IIb B.

## Figures and Tables

**Table 1:** Quantitative interface analysis of binary and quaternary complexes.

Parameter	G9-IIb A /A16	G9-IIb B /A16	Change (Δ)	Change (%)	G9-IIb A/ A16/A56/K2	G9-IIb B/ A16/A56/K2	Change (Δ)	Change (%)
Structural Metrics								
Centroid Distance (Å)	26.767	27.081	+0.314	+1.2	45.797	47.332	+1.535	+3.4
Contact Surface Area (Å^2^)								
Ligand Total	1254.50	1266.20	+11.7	+0.9	462.59	560.39	+97.8	+21.1
Ligand Polar	589.56	561.62	−27.94	−4.7	202.5	264.3	+61.8	+30.5
Ligand Nonpolar	664.98	704.64	+39.66	+6	260.09	296.1	+36.01	+13.8
Receptor Total	1236.00	1260.60	+24.6	+2	462.74	589.33	+126.59	+27.4
Receptor Polar	507.43	527.19	+19.76	+3.9	246.69	322.45	+75.76	+30.7
Receptor Nonpolar	728.57	733.45	+4.88	+0.7	216.05	275.88	+59.83	+27.7
Total BSA (Å^2^)	2490.50	2526.80	+36.3	+1.5	925.33	1149.72	+224.39	+24.3
Specific Interactions								
Hydrogen Bonds	23	28	+5	+21.7	9	6	−3	−33.3
Salt Bridges	1	3	+2	+200	0	1	+1	N/A
Pi Interactions	14	19	+5	+35.7	5	7	+2	+40

For binary complexes, ligand = G9 and receptor = A16. For quaternary complexes, ligand = G9/A16 subcomplex and receptor = A56/K2 complex. Centroid distances represent G9-A16 (binary) or G9/A16-A56/K2 (quaternary). Percentage changes for salt bridges in quaternary complexes are not applicable (N/A) due to zero baseline.

## Data Availability

All data supporting the findings of this study are available within the article and Supplementary Materials. Data S1 and Data S3 provides sequence background information, and Data S2 and Data S4 provides the complete sequences for the MPXV-I dataset and the MPXV-IIb dataset. Data S5 contains the comprehensive mutation spectrum analysis for all 11 EFC proteins across the MPXV-IIb dataset. Data S6 provides the amino acid sequences of both full-length and extracellular domains for A16, A56, G9-IIb A, G9-IIb B, and K2 proteins. Data S7 provides all AlphaFold3-predicted structural models in PDB format. Data S8 provides AlphaFold3 model confidence metrics for all predicted structures. Data S9 provides predicted monomer models of Protenix, Boltz-2, and OpenFold3 in PDB and cif format.

## Supplementary Materials

The following supporting information can be downloaded at: https://www.mdpi.com/article/10.3390/molecules31091466/s1, Figure S1: Validation of AlphaFold3 predictions by structural alignment with experimental VACV structures and local environment analysis of the M142I mutation site; Figure S2: Phylogenetic tree of 633 MPXV sequences of clade IIb; Table S1: Cross-method Cα RMSD comparison of five protein monomers predicted by AlphaFold 3, Protenix, Boltz-2, and OpenFold3; Data S1: Sequence background information of monkeypox virus clade I genetic diversity sequence dataset (*n* = 100) created from NCBI virus database; Data S2: Sequences of monkeypox virus clade I genetic diversity sequence dataset (*n* = 100) created from NCBI virus database; Data S3: Sequence background information of monkeypox virus clade IIb genetic diversity sequence dataset (*n* = 633) created from NCBI virus database; Data S4: Sequences of monkeypox virus clade IIb genetic diversity sequence dataset (*n* = 633) created from NCBI virus database; Data S5: Summary of mutation spectrum in MPXV-IIb dataset (*n* = 633); Data S6: The amino acid sequences of the complete length and extracellular domains of A16, A56, G9-IIb A, G9-IIb B, and K2 proteins; Data S7: AlphaFold3 structural models in PDB format. The archive includes: (i) five protein monomer structures (G9-IIb A, G9-IIb B, A16, A56, K2), (ii) two binary complex models (G9-IIb A/A16 and G9-IIb B/A16), and (iii) two quaternary complex models (G9-IIb A/A16/A56/K2 and G9-IIb B/A16/A56/K2); Data S8: AlphaFold model confidence metrics (pLDDT, PAE, and ipTM) for all predicted structures; Data S9: Five protein monomer models provided in PDB and CIF formats predicted using Proteinix, Boltz-2, and OpenFold3 respectively.
