# Supplementary material for: Structural Insights into the Impact of the M142I Mutation in Monkeypox Virus G9 Protein on Subcomplex Formation Revealed by AlphaFold 3 Modeling
Source: Molecules. 2026 Apr 28;31(9):1466. doi: 10.3390/molecules31091466 (PMC13165415; doi:10.3390/molecules31091466)
Supplement: Supplementary file 1 [file molecules-31-01466-s001.zip › Supplementary files/Supplement_Materials.pdf]

Supplementary Materials for

Structural Insights into the Impact of the M142I Mutation in Monkeypox Virus G9 Protein on Subcomplex Formation Revealed by AlphaFold 3 Modeling

Xudong She, Yuan Liang, Linqing Wang, Yifan Lin, Xuenan Zhang, Li Zhu, Qinghua Wu, Weiwei Xiao, Chengsong Wan, Kexin Xi, Wei Zhao, Chenguang Shen, Bao Zhang and Jianhai Yu

Correspondence to: zhaowei@smu.edu.cn (W.Z.); a124965468@smu.edu.cn (C.S.); zhangb@smu.edu.cn (B.Z.); chienhai@163.com (J.Y.)

This PDF file includes:

Figure S1. Validation of AlphaFold3 predictions by structural alignment with experimental VACV structures and local environment analysis of the M142I mutation site.

Figure S2. Phylogenetic tree of 633 MPXV sequences of clade IIb.

Table S1. Cross-method Cα RMSD comparison of five protein monomers predicted by AlphaFold 3, Protenix, Boltz-2, and OpenFold3.

Other Supplementary Materials for this manuscript include the following:

Data S1. Sequence background information of monkeypox virus clade I genetic diversity sequence dataset (n=100) created from NCBI virus database.

Data S2. Sequences of monkeypox virus clade I genetic diversity sequence dataset (n=100) created from NCBI virus database.

Data S3. Sequence background information of monkeypox virus clade IIb genetic diversity sequence dataset (n=633) created from NCBI virus database.

Data S4. Sequences of monkeypox virus clade IIb genetic diversity sequence dataset (n=633) created from NCBI virus database.

Data S5. Summary of mutation spectrum in MPXV-IIb dataset (n=633).

Data S6. The amino acid sequences of the complete length and extracellular domains of A16, A56, G9-IIb A, G9-IIb B, and K2 proteins.

Data S7. AlphaFold3 structural models in PDB format. The archive includes: (i) five protein monomer structures (G9-IIb A, G9-IIb B, A16, A56, K2), (ii) two binary complex models (G9-IIb A/A16 and G9-IIb B/A16), and (iii) two quaternary complex models (G9-IIb A/A16/A56/K2 and G9-IIb B/A16/A56/K2).

Data S8. AlphaFold3 model confidence metrics (pLDDT, PAE, and ipTM) for all predicted structures.

Data S9. Five protein monomer models provided in PDB and CIF formats predicted using Proteinix, Boltz-2, and OpenFold3 respectively.

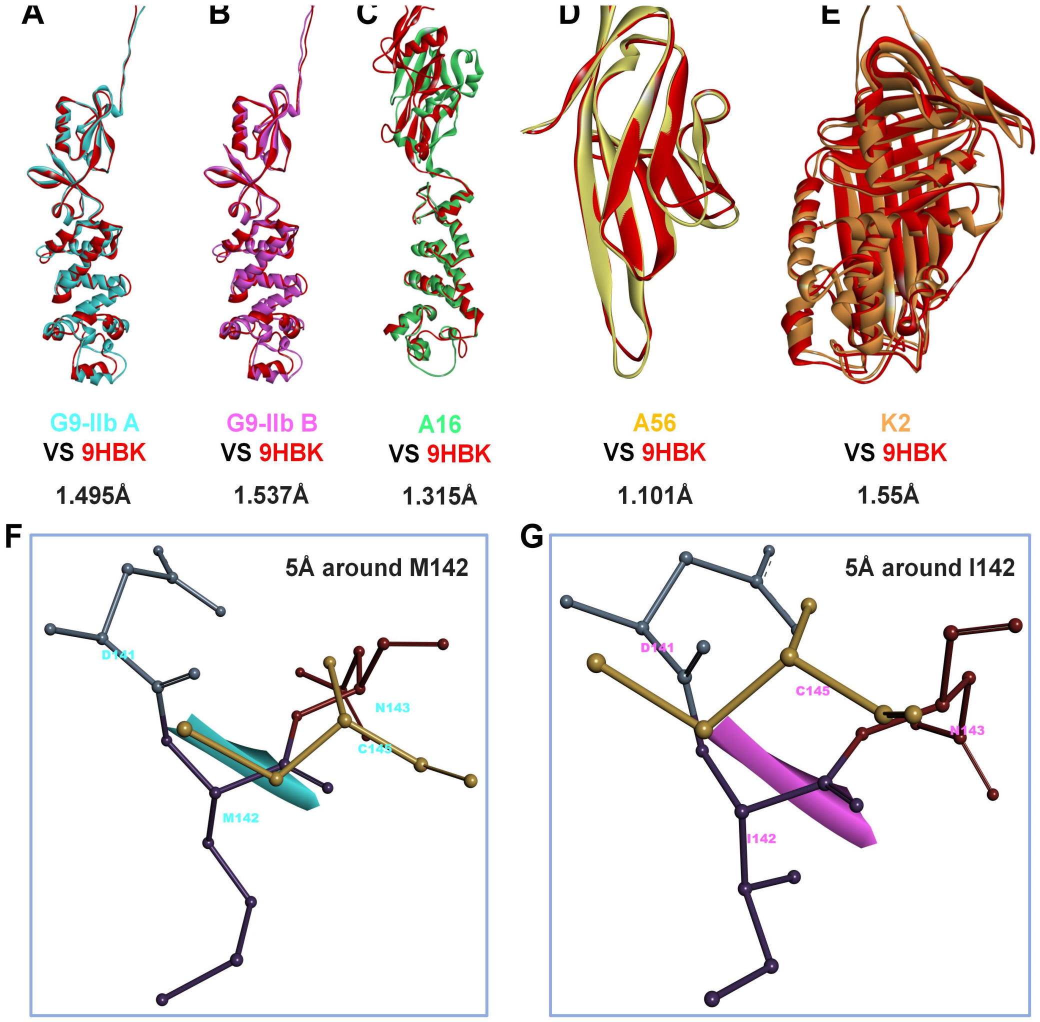

Figure S1. Validation of AlphaFold3 predictions by structural alignment with experimental VACV structures and local environment analysis of the M142I mutation site.

(A-E) Superposition of predicted monomer structures (cyan: G9-IIb A; magenta: G9-IIb B; green: A16; yellow: A56; brown: K2) onto corresponding chains from the vaccinia virus crystal structure (PDB: 9HBK, shown in red). RMSD values for Cα atoms are indicated below each panel. (F-G) Close-up view of the 5Å neighborhood surrounding residue 142 in G9-IIb A (M142, F) and G9-IIb B (I142, G). Side chains of neighboring residues (D141, N143, C145) are displayed as sticks, with the central residue (M142/I142) highlighted in cyan and magenta, respectively. No steric clashes are observed between I142 and surrounding residues.

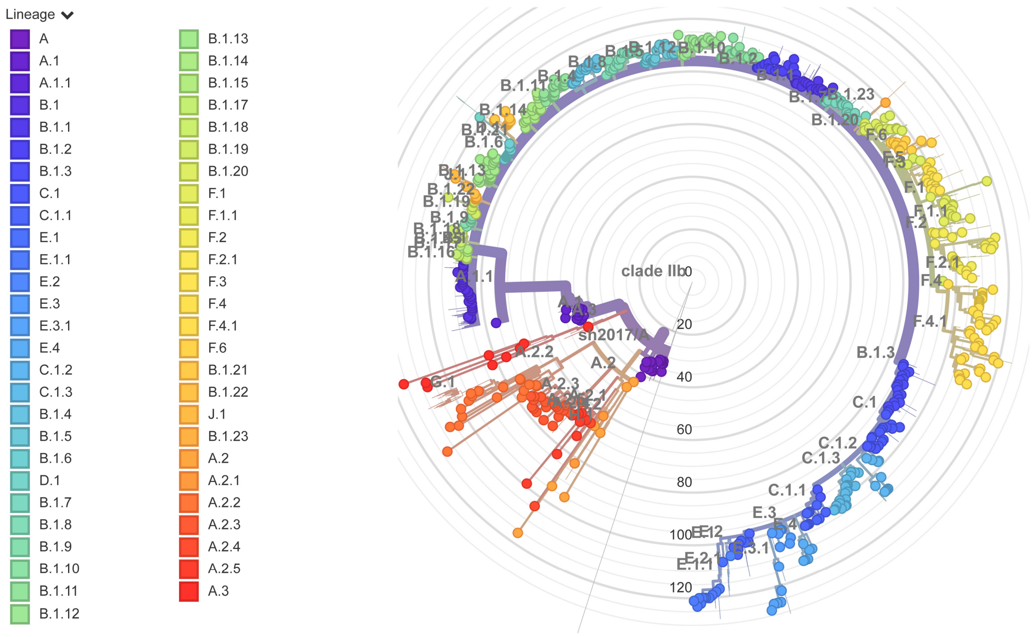

Figure S2. Phylogenetic tree of 633 MPXV sequences of clade IIb.

The phylogenetic tree was generated using NextClade v2.14.1 online server for lineage assignment purposes only. The server outputs results in SVG format; therefore, tree-viewing compatible files (e.g., Newick) are not available.

Table S1. Cross-method Cα RMSD comparison of five protein monomers predicted by AlphaFold 3, Protenix, Boltz-2, and OpenFold3.

	AlphaFold 3				
	G9-IIb A	G9-IIb B	A16	A56	K2
Protenix	1.17Å	1.621Å	0.783Å	0.933Å	0.635Å
Boltz-2	0.903Å	1.184Å	0.895Å	0.794Å	1.354Å

OpenFold3	0.849Å	0.778Å	0.679Å	1.638Å	1.169Å
